# Assessment of nerve conduction velocity slowing and its association with the severity of diabetic polyneuropathy, duration of diabetes and glycemic control in diabetic patients

**DOI:** 10.12669/pjms.41.3.10397

**Published:** 2025-03

**Authors:** Saba Zaidi, Ayesha Abdul Samad

**Affiliations:** 1 Saba Zaidi, FCPS Neurology Liaquat National Hospital, Karachi Pakistan; 2 Ayesha Abdul Samad, MBBS Liaquat National Hospital, Karachi Pakistan

**Keywords:** Glycemic control, Male, Glycated hemoglobin, Diabetic Neuropathy, Nerve conduction study

## Abstract

**Objective:**

This study aimed to investigate the nerve conduction velocity in diabetic patients and its association with diabetes duration, control and severity of diabetic polyneuropathy.

**Methods:**

This prospective, observational study involved a hundred and thirty-nine patients who underwent nerve conduction studies in the Neurology Department at Liaquat National Hospital during the years June 2023 till May 2024. In all patients, the medical history was taken by direct interview regarding demographics, diabetes duration and recent HbA1c. For the assessment of diabetic polyneuropathy, the NDS-neuropathy disability score was used. The acquired data was entered into SPSS Statistics software for analysis of significant associations between these variables. Statistical significance was defined as a P-value below 0.05.

**Results:**

In this study, we investigated 139 patients with a mean age of 62.20±12.03 years, comprising 60.4% males. The mean HbA1c was 7.46±1.09%, and the mean duration of diabetes was 10.78±7.75 years. Most patients (75.5%) had poor glycemic control, with 92.8% having Type-II diabetes. The mean neuropathy disability score was 5.63±2.39, with 80.6% of patients experiencing neuropathy. Nerve conduction velocity slowing was detected in 54.7% of the patients. Notably, significant associations were found between nerve conduction velocity slowing and diabetes duration (p=0.019), and neuropathy disability score (p=0.000).

**Conclusion:**

Our findings indicate that male gender, poor glycemic control, and a longer duration of diabetes are associated with slowing of nerve conduction velocity. Additionally, the severity of neuropathy, as measured by the Neuropathy disability score, further strengthens these associations, highlighting its significance in assessing diabetic neuropathy progression.

## INTRODUCTION

Diabetic neuropathy serves as the predominant microvascular complication of almost half of the people affected by both type-1 and type-2 diabetes mellitus. While it can already be present at the time of the diagnosis of T2DM, it appears almost 10 years after the onset of the disease in the case of T1DM. This can greatly reduce the quality of life of the patients as a consequence of sensory loss, fall-related injury, disabling neuropathic pain, gait instability, foot ulceration and amputation. The latter also contributes as a major health and economic burden as lower limb amputation rates related to diabetes are increasing.^1^ Therefore early diagnosis and screening of signs and symptoms of DPN is crucial because it provides an opportunity to identify DPN at the initial asymptomatic stages to prevent further advancement.^2^ Pathogenesis of diabetic polyneuropathy includes several factors such as persistent hyperglycemia, oxidative stress and autoimmune-mediated nerve destruction which can result in axonal degeneration and gradual nerve fibre loss.^3^

Several risk factors of DPN have been identified in both prospective epidemiologic and cross-sectional studies among which poor glycemic control reflected by increased glycated hemoglobin (also known as HbA1c) is the most important factor. Other important factors identified are the duration of diabetes as well as demographic factors including height, age, and male sex. Certain studies have indicated that hypertension, smoking, elevated triglyceride low HDL cholesterol, and other risk factors for atherosclerosis may also be associated with an increased risk of diabetic polyneuropathy.^4^ According to a consensus report by the American Academy of Neurology, the most accurate diagnosis of distal symmetric polyneuropathy is achieved through a combination of symptoms, signs, and results from electrophysiological tests, however, nerve conduction studies (NCS) serve as the gold standard for diagnosing DPN.^5^

The parameters used mainly in NCS are amplitudes, latencies and nerve conduction velocities.^6^ These parameters assist in distinguishing between demyelinating neuropathy and axonal degeneration because NCV reflects changes in myelin, while amplitude indicates axonal changes and the overall condition of nerve fibers.^7^ In DPN, sensory nerve action potentials (SNAP) identify the function of sensory nerve fibers from the peripheral receptors in the skin to the dorsal root ganglia while compound muscle action potentials (CMAP) explain the activity of motor nerve fibers, from the anterior horn cell to their termination at muscle fibers.

This description allows us to distinguish the underlying nerve pathophysiology, characterized by either loss of axons or demyelination.^4^ The purpose of this comprehensive study was to identify the prevalence of conduction velocity slowing in patients with diabetic polyneuropathy and to draw its association with longer duration of diabetes, poor glycemic control and severity of clinical symptoms as assessed by neuropathy disability score.

## METHODS

The present research was an observational, prospective study conducted on a hundred and thirty-nine diabetic patients who were seen in Liaquat National Hospital, neurophysiology department over twelve months from June 2023 till May 2024.

### Inclusion & Exclusion Criteria:

After elucidating the research methodology and obtaining informed consent from patients, they willingly enrolled in the current study. The exclusion criteria were individuals diagnosed with neurological or peripheral neuropathic disorders attributed to vitamin B12 deficiency or hypothyroidism, as well as those with a documented history of alcohol or drug abuse. Additionally, patients with pre-existing chronic illnesses, such as autoimmune diseases or cancer, which may have implications for nerve function, are also excluded from participation.

The patients’ medical histories were documented, followed by comprehensive physical and neurological examinations. The questionnaire utilized in this study encompassed inquiries regarding demographics, duration of diabetes, complications related to diabetes, neuropathy severity assessment and smoking history. Neuropathy disability score includes temperature, vibration sensation, pinprick and ankle jerk. The last part of the study is devoted towards the nerve conduction velocity measurement.

The nerve conduction study was conducted by skilled neurophysiology technicians who underwent training in standardized protocols to ensure uniformity and accuracy throughout data acquisition sessions. Recordings were carried out under controlled temperature conditions (32-36°C), with meticulous attention to distance measurements. For the neuropathy protocol, specific nerves were selected, including the non-dominant median nerve, dominant peroneal nerve, and bilateral sural nerves. Parameters assessed included nerve conduction velocities, compound muscle action potentials (CMAPs), sensory nerve action potentials (SNAPs), and distal latencies across all designated nerves. Simultaneously, electromyography (EMG) was performed to detect the presence of neurogenic motor unit action potentials (MUAPs).

### Ethical Approval:

It was obtained on 12^th^ June 2024, from the Hospital’s Ethical review committee with reference number # 1047-2024-LNH-ERC.

### Statistical Analysis:

Data analysis was done by using IBM SPSS statistics version 27. Mean and standard deviation were calculated for quantitative variables. Frequency and percentage were reported for qualitative variables. Chi-square/fisher exact tests were applied to determine the association between qualitative variables. Odds were calculated by binary logistic regression p-value less than 0.05 were considered as significant.

## RESULTS

**Fig.1:**
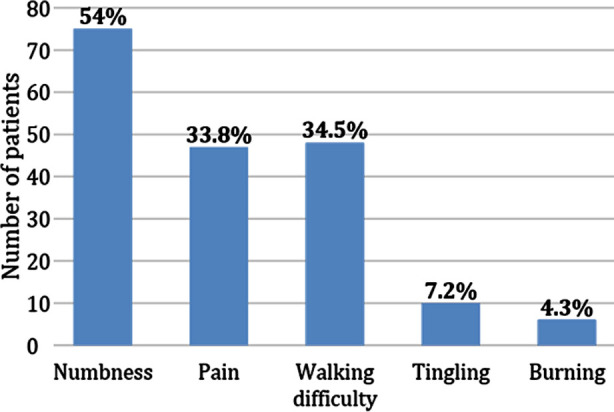
Frequency of symptoms of diabetic polyneuropathy.

The current study included 139 patients in total, with a mean age of 62.20±12.03 years and 60.4% males and 39.6% females. The mean HbA1c and duration of diabetes were 7.46±1.09% and 10.78±7.75 years, respectively. 75.5% of the patients had poor glycemic control whereas, 92.8% with Type-II diabetes and 7.2% with Type-I diabetes. Mean neuropathy disability score of 5.63±2.39 whereas, 80.6% of the patients had neuropathy. 14.4% of patients were on medication for diabetic neuropathy and 35.3% were smokers. There were 97.8% of patients with normal latencies, 57.6% of patients had neurogenic motor unit action potentials on EMG, and 95.7% of patients had low CMAPs (compound muscle action potential) and SNAPs (sensory nerve action potential). In our investigation, nerve conduction velocity slowing was detected in 54.7% of the patients. As seen in Fig.1, the majority of patients report symptoms of numbness (n=75) and pain (n =47).

**Fig.2:**
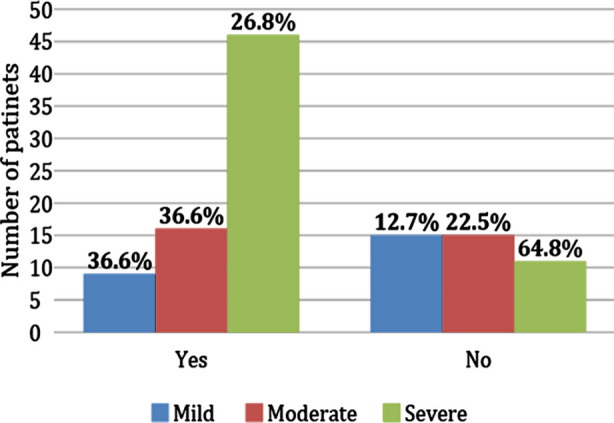
Comparison of Neuropathy severity in patients with or without NCV slowing.

There was significant association of nerve conduction velocity slowing with diabetes duration (p=0.019), Neuropathy disability score (p=0.000) and neurogenic motor units on EMG (p=0.04) while no significant association were found with gender (p=0.077), age (p=0.514), HbA1C level (p=0.815), comorbidities other than diabetes (p=0.345), smoking status (p=0.941), low CMAPs and SNAPs (p=0.410), and latencies (p=1.000). Table-I displays the comprehensive results of the associations and Fig.2 demonstrates the relationship of Neuropathy disability score with nerve conduction velocity slowing.

**Table-I:** Association of Nerve conduction velocity slowing with demographics.

	Nerve conduction velocity slowing		P-Value

	Yes	No	
***Gender***		
Male	51 (67.1)	33 (52.4)	0.077
Female	25 (32.9)	30 (47.6)	
***Age(years)***			
31-40 years	5 (6.3)	4 (6.3)	0.514
41-50 years	5 (7.9)	5 (7.9)	
51-60 years	11 (17.5)	11 (17.5)	
61-70 years	26 (41.3)	26 (41.3)	
>70 years	17 (27)	17 (27)	
***HbA1C Level (%)***			
Poor glycemic control ≥7)	58 (76.3)	47 (74.6)	0.815
Good glycemic control (<7)	18 (23.7)	16 (25.4)	
***Diabetes duration (years)***			
≤5 years	14 (18.4)	25 (39.7)	0.019*
6-10 years	33 (43.4)	22 (34.9	
>10 years	29 (38.2)	16 (25.4)	
***Neuropathy disability***			
Yes	71 (93.4)	41 (65.1)	0.000*
No	5 (6.6)	22 (34.9)	
***Chronic disease***			
Yes	41 (53.9)	39 (61.9)	0.345
No	35 (46.1)	24 (38.1)	
***Smoking Status***			
Smoker	27 (35.5)	22 (34.9)	0.941
Non-Smoker	49 (64.5)	41 (65.1)	
***CMAPs and SNAPs-Low***			
Present	59 (93.7)	74 (97.4)	0.410
Absent	4 (6.3)	2 (2.6)	
***EMG neurogenic***			
Neurogenic	52 (68.4)	28 (44.4)	0.004*
Normal	24 (31.6)	35 (55.6)	
Latencies			
Prolong	2 (2.6)	1 (1.6)	1.000
Normal	74 (97.4)	62 (98.4)	

Chi-square/ fisher exact test was applied, p-value<0.05 considered as significant,

* Significant at 0.05 levels.

According to univariate logistic regression analysis, patients with good glycemic control when compared with poor control are more likely to exhibit nerve conduction velocity slowing (OR=1.097, p=0.815). Regarding the duration of diabetes and nerve conduction velocity (NCV) slowing; Individuals with diabetes for five years or less (OR = 0.309, p = 0.010) are less likely to have slowed NCV compared to those with diabetes for more than 10 years.

Similarly, individuals with diabetes duration between 6-10 years (OR = 0.828, p = 0.649) do not show a significant difference in the likelihood of NCV slowing compared to those with diabetes for more than 10 years. This suggests that NCV slowing is more strongly associated with a diabetes duration exceeding 10 years. Low CMAPs and SNAPs in the Nerve conduction study showed a high likelihood of the presence of NCV slowing (OR=2.508, p=0.298). Compared to patients with normal EMG, those with neurogenic EMG are more likely to exhibit nerve conduction velocity slowing (OR=2.708, p=0.005). Individuals with NCV slowing are more likely to have normal latencies. (OR=1.676, p=0.676). Table-II.

**Table-II:** Odds for Nerve conduction velocity slowing presence.

	Un-adjusted		Adjusted	

	p-value	odds ratio (95% CI)	p-value	odds ratio (95% CI)
***Gender***				
Male	0.079	1.855(0.932-3.690)		
Female		Ref		
***Age(years)***				
31-40 years	0.935	1.062(0.245-4.599)		
41-50 years	0.407	1.700(0.486-5.953)		
51-60 years	0.445	1.468(0.548-3.930)		
61-70 years	0.453	0.719(0.304-1.700)		
>70 years		Ref		
***HbA1C Level (%)***				
Poor glycemic control ≥7)	0.815	1.097(0.505-2.382)		
Good glycemic control (<7)		Ref		
***Diabetes duration (years)***				
≤5 years	0.010	0.309 (0.126-0.756)		
6-10 years	0.649	0.828(0.366-1.869)		
>10 years		Ref		
***Neuropathy disability***				
Yes	0.000	7.620(2.681-21.652)	0.000	7.183(2.482-20.788)
No		Ref		Ref
***Chronic disease***				
Yes	0.345	0.721(0.365-1.423)		
No		Ref		
***Smoking Status***				
Smoker	0.941	1.027(0.510-2.066)		
Non-Smoker		Ref		
***CMAPs and SNAPs-Low***				
Present	0.298	2.508(0.444-14.171)		
Absent		Ref		
***EMG neurogenic***				
Neurogenic	0.005	2.708(1.354-5.418)	0.014	2.518(1.205-5.263)
Normal		Ref		Ref
***Latencies***				
Prolong	0.676	1.676(0.148-18.921)		
Normal		Ref		

Binary logistic regression was applied, p-value<0.05 considered as significant., *Significant at 0.05 levels.

## DISCUSSION

In our study, 54.7% (n=75/139) of the patients exhibited a slowing of nerve conduction velocity (NCV), highlighting the high prevalence of this neurophysiologic finding among participants with diabetes. Although the literature on the assessment of this specific parameter among diabetics is limited, our findings align with the existing studies, suggesting that more than half of diabetic patients may experience some degree of nerve conduction impairment.^8^-^10^

It has been well established that diabetic polyneuropathy primarily manifests as axonal, as evidenced by low sensory nerve action potentials (SNAPs) and compound muscle action potentials (CMAPs). In our selected cases (n=133, 95.7%), low motor and sensory amplitudes were consistent with expectations for diabetic polyneuropathies. The presence of nerve conduction velocity (NCV) slowing in our patients raises concerns for concurrent chronic inflammatory demyelinating neuropathies (CIDP).

However, our findings contradict this concern, as 97% (n=136) of our patients exhibited normal distal latencies in the selected nerves, which do not fulfil the diagnostic criteria for CIDP. The primary explanation for this isolated observed slowing of nerve conduction velocity might be a severe axonal injury, resulting in some effects on saltatory conduction through myelin. Unfortunately, due to inconsistencies in data collection, we were unable to precisely quantify the exact velocities. Consequently, we cannot comment on the degree of slowing. This limitation underscores the critical need for standardized data collection methods in future studies to effectively assess nerve conduction parameters in diabetic patients.

Age is consistently shown to correlate with reduced NCV, indicating that older patients are more likely to experience severe conduction slowing.^11^ But in our studies it failed to attain a significant statistical value (p=0.514). This can be because the majority of our patients were between the ages of 51-70 years, potentially reducing the variability needed to detect a significant effect. This range may not provide enough variability to detect age-related differences in NCV, which are typically more noticeable when comparing a broader age span.

Our study identified a significant correlation between the duration of diabetes and NCV (nerve conduction velocity) slowing (p=0.019). This finding is consistent with a 2017 cross-sectional survey, which also determined that a longer duration of diabetes is a significant risk factor for slowing NCV^12^ Similarly, another study conducted by Charles M et al, demonstrated similar findings.^10^ Glycemic control continues to be a key predictor for the development of diabetic peripheral neuropathy (DPN) and subsequent changes in (nerve conduction velocity) NCV. A study examining the immediate impact of glycemic control on nerve conduction found that glycemic control rapidly influences nerve conduction speed, as demonstrated in our research.^13^

Other studies conducted on patients with T1DM and T2DM have also noted that poor glycemic control accelerates the reduction in nerve conduction velocity in diabetic patients.^14^,^15^ 75.5% (n=105) of the patients in our research had poor glycemic control, indicated by an HbA1c level of >7%. Despite the majority of participants having uncontrolled diabetes, we did not find a statistically significant association between HbA1c levels and NCV (nerve conduction velocity) slowing. The reasons might be that we took a recent HbA1c level, which reflects glycemic control over the previous three months only, while most of our patients had diabetes for more than five years (n=55, 39.6%), with the majority receiving treatment for their diabetes control (n=128, 92.1%). Additionally, the lack of a significant association could be due to the influence of long-standing diabetes and varying treatment adherence over the years, which might not be fully captured by a single recent HbA1c measurement.

Furthermore, amputations are on the rise, underscoring the need to reconsider our current thresholds for glycemic control. We may need to adopt a higher cut-off for HbA1c or employing other parameters for glycemic control, such as Continuous Glucose Monitoring (CGM), which provides more comprehensive and dynamic insights into glucose levels over time. NDS (Neuropathy Disability Score) is a clinical tool used to assess the severity of diabetic neuropathy. It classifies patients into four groups: no, mild, moderate, and severe neuropathy.^16^ In our study, most patients had an NDS of eight, indicating severe neuropathy, with 57 out of 139 patients (50.9%) falling into this category. Simultaneously, 54% (n=75) of the patients reported numbness, which is consistent with existing literature, where numbness is commonly reported as a primary complaint among neuropathic patients.

Neuropathy disability has a significant association with nerve conduction velocity (NCV) slowing, and the severity of neuropathy is often quantified by the NDS.^17^ It was concluded in a cross-sectional study that nerve conduction velocity declines as neuropathy worsens with the progression of type-2 diabetes mellitus, and this pattern is observed in both motor and sensory nerves.^18^ Similarly, our study also found a significant association between NCV slowing and NDS, with a p=0.000. The significant association is likely because NDS comprehensively assessed multiple aspects of neuropathy severity, including sensory and motor deficits, which are closely related to nerve conduction abnormalities. This multidimensional assessment makes NDS a robust tool for capturing the extent of neuropathic damage that directly correlates with NCV measurements.

### Strength of the study:

Our study possesses several strengths that enhance its contribution to the existing body of literature. Firstly, it includes a substantial number of patients, providing ample data for robust analysis. Additionally, the examination of multiple variables, including glycemic control, diabetes duration, and neuropathy severity, adds depth to our understanding of the factors associated with nerve conduction velocity (NCV) slowing in diabetic patients. Despite these strengths, certain areas warrant further exploration. Notably, while our study focused on identifying factors associated with NCV slowing, it did not investigate the prevalence of chronic inflammatory demyelinating polyneuropathy (CIDP) among patients with diabetic peripheral neuropathy (DPN). This presents an important area for future research, as early identification of CIDP in diabetic patients may significantly influence treatment decisions and outcomes. Future studies should aim to investigate the prevalence and clinical implications of CIDP in DPN populations. Addressing these gaps will further refine the diagnostic and therapeutic approaches for managing neuropathy in diabetic patients.

### Limitation:

It includes a single-center study, the generalizability of our findings to a larger population may be limited. Furthermore, the observational study design precludes insights into the progression of NCV slowing over time. A longitudinal study design would be a more appropriate approach to track changes and understand the progression of NCV slowing over an extended period. Another drawback is that we did not use quantitative mean values to assess the degree of NCV slowing. Therefore, we propose that future research should focus on quantifying NCV slowing and exploring alternative parameters for assessing glycemic control beyond HbA1c levels like continuous glucose monitoring.

## CONCLUSION

Our findings indicate that male gender, poor glycemic control, and a longer duration of diabetes are associated with slowing of nerve conduction velocity. Additionally, the severity of neuropathy, as measured by the Neuropathy disability score (NDS), further strengthens these associations, highlighting its significance in assessing diabetic neuropathy progression.

### Author’s Contribution:

SZ: Substantial contributions to conception & design, or acquisition of data, analysis & interpretation of data. Accountable for all aspects of the work in ensuring integrity of the study.

AAS: Drafting the article , revising it critically for important intellectual content and final approval of the version to be published.
